# Interventions for promoting patients’ adherence to 14-day primaquine treatment in a highly malaria-endemic township in Myanmar: A qualitative study among key stakeholders

**DOI:** 10.21203/rs.3.rs-3312278/v1

**Published:** 2023-09-06

**Authors:** Kyawt Mon Win, Pyae Linn Aung, Zau Ring, Nay Yi Yi Linn, Myat Phone Kyaw, Wang Nguitragool, Liwang Cui, Jetsumon Sattabongkot, Saranath Lawpoolsri

**Affiliations:** Mahidol University; Mahidol University; Ministry of Health; Ministry of Health; Myanmar Health Network Organization; Mahidol University; University of South Florida; Mahidol University; Mahidol University

**Keywords:** Myanmar, Intervention, Plasmodium vivax, Primaquine, Qualitative research, Treatment adherence, Compliance

## Abstract

**Background:**

*Plasmodium vivax* malaria is considered a major threat to malaria eradication. The radical cure for *P. vivax* malaria normally requires a 14-day administration of primaquine (PQ) to clear hypnozoites. However, maintaining adherence to PQ treatment is a significant challenge, particularly in malaria-endemic rural areas. Hence, this study aimed to formulate interventions for promoting patients’ commitment to PQ treatment in a highly malaria-endemic township in Myanmar.

**Methods:**

A qualitative study was conducted in Waingmaw Township in northern Myanmar, where *P. vivax* malaria is highly endemic. Key stakeholders including public health officers and community members participated in focus group discussions (FGDs) and in-depth interviews (IDIs) in September 2022. Data were collected using validated guidelines, translated into English, and visualized through thematic analysis.

**Results:**

Responsible individuals from different levels of the Myanmar National Malaria Control Program participated in the IDIs. Most of them reported being aware of the markedly increasing trend of *P. vivax* and the possibility of relapse cases, especially among migrants who are lost to follow-up. Workload was a key concern surrounding intervention implementation. The respondents discussed possible interventions, such as implementing directly observed treatment (DOT) by family members, piloting a shorter PQ regimen, expanding the community’s malaria volunteer network, and strengthening health education activities using local languages to promote reasonable drug adherence. FGDs among community members revealed that although people were knowledgeable about malaria symptoms, places to seek treatment, and the use of bed nets to prevent mosquito bites, most of them still preferred to be treated by quack doctors and rarely used insecticide-treated nets at worksites. Many often stopped taking the prescribed drugs once the symptoms disappeared. Nevertheless, some respondents requested more bed nets to be distributed and health promotion activities to be conducted.

**Conclusion:**

In rural areas where human resources are limited, interventions such as implementing family member DOT or shortening PQ regimens should be introduced to enhance the radical cure for the *P. vivax* infection. Disseminating information about the importance of taking the entire treatment course and emphasizing the burden of relapse is also essential.

## Background

Despite a significant reduction in reported annual malaria cases in Myanmar, statistics from 2021 showed that 88% of the country’s malaria cases were from the Greater Mekong Subregion (GMS) [1]. That year, 81.4% of the 79,001 confirmed malaria cases from the country’s 330 townships were caused by the *Plasmodium vivax* infection [1]. Malaria incidence in Myanmar decreased because access to diagnosis and treatment services expanded through basic health staff (BHS) and village health volunteers (VHVs), especially in rural and highly malaria-endemic areas [2, 3]. Currently, *P. vivax* has become the predominant cause of *Plasmodium* infection in the country. The radical cure for *P. vivax* malaria requires a 14-day primaquine (PQ) treatment to eliminate hypnozoites in the human liver [4]. However, patients’ compliance with such a regimen is a major challenge [5, 6]. According to a recent report, approximately 20% of malaria cases presented recurrent or relapse episodes, most of which occurring within one year after the initial infection [7]. Because relapse infections can help sustain the infectious parasite reservoir in the community, they are an important impediment to the eradication of malaria [8].

In 2011, the Myanmar National Malaria Control Program (NMCP) introduced PQ to the National Antimalaria Treatment Policy, which recommended that a 3-day chloroquine together with a 14-day PQ (0.25mg/kg/day) be given to patients with *P. vivax* malaria by BHS without glucose 6 phosphate dehydrogenase (G6PD) testing. In areas where health facilities are limited in handling severe hemolysis, another PQ regimen (0.75mg/kg/week for 8 weeks) should be prescribed by VHVs [9]. In July 2018, to promote treatment adherence, the guidelines were modified to allow VHVs to administer the 14-day PQ (0.25mg/kg/day) to patients with *P. vivax* instead of the weekly regimen. Still, the practice of administering the G6PD test to every patient is not yet recommended in Myanmar. Patients undergoing the 14-day PQ treatment are instructed to check their urine color as a sign of hemolysis; if their urine turns black or red, they must stop PQ medication and seek further hemolysis management at the nearest health centers [9].

Because *P. vivax* transmission is a major threat to malaria eradication in the country, enhancing activities focusing on the radical cure for *P. vivax* is a top priority under the Myanmar National Strategic Plan for Malaria Elimination (2021–2025) [10]. Under the current intervention program, adherence to the 14-day PQ regimen is estimated to be low. A study conducted in late 2015 found that only 46% of patients treated by VHVs complied with PQ treatment [11]. This highlights a need for new approaches to improve adherence; however, to be considered effective, new interventions require several considerations, including financial and human resources, community acceptance and participation, and social and local beliefs. Effective interventions must obtain inputs from frontline health workers as well as the community, and the challenges that prevent patients from strictly following treatment instructions must be explored and addressed. One study in Myanmar recommended that qualitative studies be conducted on factors that drive patient adherence to PQ [11].

Myanmar is composed of 14 states and regions and 1 union territory. Each locality is different in terms of geographical features, displacement patterns of people, movement of migrants, and malaria situation including vector bionomics. Consequently, disparities in malaria prevalence can be observed across regions. For instance, in 2021, the states of Kachin, Kayin, and Rakhine reported the highest malaria cases in the country. Formulating the most effective intervention to reduce the number of patients in a particular area involves tailoring the intervention to the nature of its location, demographics, and predominant malaria species [12]. Therefore, optimal interventions for each setting must include inputs from frontline health workers and the community. This study aims to examine the various factors that influence *P. vivax* malaria patients’ compliance with PQ treatment in a township in northern Myanmar. Ultimately, our goal is to create a novel intervention package that can improve adherence among these patients.

## Methods

### Study design

This qualitative study includes in-depth interviews (IDIs) with healthcare providers from all administrative levels of the NMCP (central, state, township, and village) and focus group discussions (FGDs) with community members to collect their perspectives, views, and inputs regarding *P. vivax* malaria treatment and treatment adherence.

### Study site

This study was conducted in Kachin State in northern Myanmar, which has a population of 1.7 million. Kachin State accounted for 36% of the nationwide reported *P. vivax* cases in 2020. Of the total malaria cases reported in this state, more than 90% were caused by *P. vivax*. Malaria infection is present throughout the year but peaks in May–August.

**Fig. 1:**
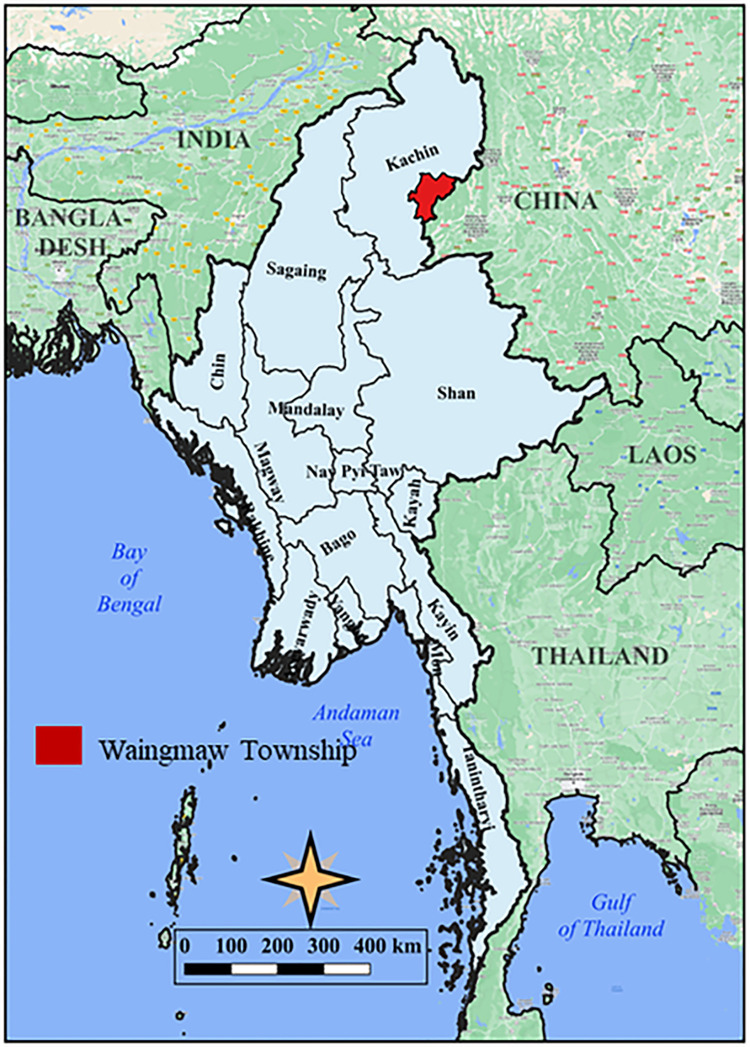
Map locating the study township in northern Myanmar

**Fig. 2:**
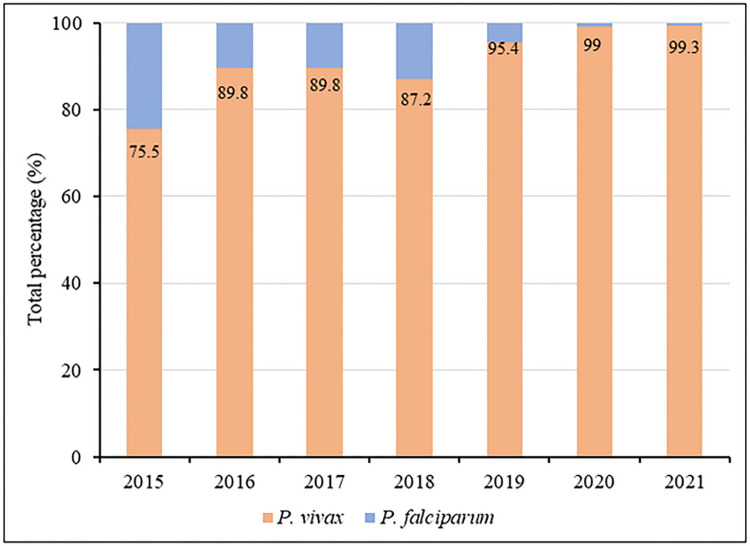
Malaria species composition in Waingmaw Township (2015–2021)

Waingmaw Township, located near the China–Myanmar border (Fig. 1), was purposively selected as the study site for having the highest incidence of *P. vivax* among the 18 townships of Kachin State. In 2020, Waingmaw Township had a total of 197 trained VHVs serving a population of approximately 100,000 across its 213 malaria-endemic villages [13]. One-third of the township is covered by forest vegetation with an average annual rainfall of 98 cm. Its annual parasite incidence was 30 per 1,000 population in 2021, of which 99.4% is attributed to *P. vivax* (Fig. 2). The township reported 5,242 and 7,953 *P. vivax* cases in 2020 and 2021, respectively.

In addition, three villages were purposively selected for the FGDs based on their high *P. vivax* burden and accessibility (security reasons).

### Study population and samples

The study population included malaria program staff from all levels of administration, residents from ethnic majority and minority groups in selected villages, mobile and migrant populations working as seasonal agricultural laborers, and forest-related workers.

**Fig. 3:**
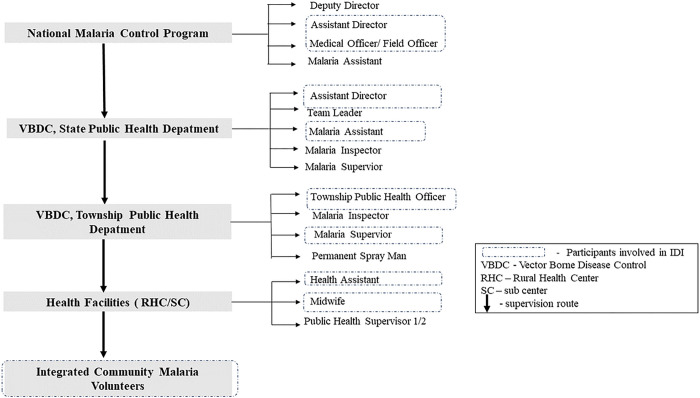
Myanmar malaria control program’s lineup from village level up to central level

To explore the perceptions of different stakeholders regarding the challenges to malaria treatment and its adherence, 10 IDIs were held at the central, state, township, and village levels (Fig. 3). Eligible participants included males or females aged more than 18 years and with more than 1 year of experience in malaria prevention and control activities in their current positions.

Also, four FGDs were conducted among community members to explore their overall malaria knowledge, perceptions, preventive practices, and suggestions for further activities in the malaria program. One FGD was held in each village except for one large village that required two FGDs. A total of six community members were invited to participate in each FGD. These community members included males and females in different occupations to capture different views, but the FGDs prioritized community members who have resided in the selected villages for at least two years and have contracted malaria at least once in the last two years. Village administrative personnel, religious leaders, or older villagers who could lead the discussion and whose voice may overpower others were excluded.

### Data collection

Guidelines for both IDIs and FGDs were developed in English based on the literature [14] and other relevant studies [11, 15]. These guidelines were translated into Burmese with the help of malaria experts from the NMCP through backtranslation. Two guidelines were written separately for healthcare providers and community members. For the IDIs, semi-structured interviews were conducted to explore healthcare providers’ operational challenges, barriers, and difficulties in implementing the National Malaria Treatment Guidelines. Meanwhile, the FGDs explored their knowledge of malaria, their perception of its susceptibility and severity, the benefits of and barriers to PQ treatment, and access to information on antimalarial medications and treatment adherence among community members.

Three data collection assistants from the central NMCP were voluntarily recruited. One day before the lead researcher gathered data, orientation sessions on data collection procedures, ethical standards, and the recording of responses were organized. An IDI lasted 20 minutes while a FGD took 1 hour. Audio recordings and handwritten notes were taken during the interviews. The IDIs and FGDs were held in secure locations, such as offices or private rooms. Informed consent was obtained before the interviews.

### Data analysis

Qualitative data from both IDIs and FGDs were independently translated into English by KMW and PLA and simultaneously transcribed in verbatim. The translations were cross-checked for accuracy by SL and ZR. The findings underwent deductive and inductive thematic analysis, and the constructed themes were shared with all authors and amended accordingly. All authors agreed on the finalized themes. Qualitative data analysis was performed with the help of NVivo version 12.

### Ethical consideration

The study protocol was reviewed and approved by the institutional review boards of the University of Public Health, Yangon, Myanmar (UPH-IRB: 2022/Research/6), and the Ethics Committee for Human Research Study, Faculty of Tropical Medicine, Mahidol University, Bangkok, Thailand (MUTM 2022–064-01). The respondents were informed about the study and signed informed consent forms before participating in the interviews or discussions.

## Results

### Participant demographics

The IDIs were conducted with 10 focal persons (3 females and 7 males) representing the different levels of the malaria program. This respondent group had a mean age of 46.7 years (SD 8.7 years). Their working experience in malaria ranged from 5 to 20 years. Two designated NMCP focal persons from the central level were responsible for various aspects, including program management, monitoring and evaluation, logistic management, and supervision. Two respondents from the Vector-Borne Disease Control Unit, representing the State Public Health Department, were accountable for the prevention and control of all vector-borne diseases in their state. Six public health officers at the township and village levels served as key management personnel responsible for overseeing all diseases in their respective areas.

Additionally, 4 FGDs were conducted with 24 community members (8 females, 16 males) from 3 malaria-endemic villages in Waingmaw Township. These respondents had a mean age of 34 years (SD 9.8 years). More than half of the respondents (63%) were engaged in forest-related work, such as in banana plantations; 16% were employed in their villages; and 20% were dependents or housewives.

### In-depth interviews with malaria healthcare providers

From the IDIs, four themes emerged reflecting the importance of the malaria burden and the challenges faced by the current antimalarial treatment as well as recommendations to improve the malaria control program.

### IDI theme I: The overall malaria situation

All participants from different levels of the malaria program acknowledged a significant decline in malaria prevalence throughout the country due to the expanded implementation of intensive malaria prevention and control activities by various funding agencies. Central level healthcare officials emphasized the emerging trend of malaria cases reported in specific townships, which may be influenced by both the influx of the population due to the ongoing economic crisis and changes in environmental factors favoring vector proliferation. While malaria cases had predominantly involved migrant workers, in 2021, Kachin State reported a substantial number of locally transmitted cases, particularly affecting younger age groups. Malaria persists throughout the years in its villages, with the highest caseloads occurring during the rainy season (May–October). While migrant populations have been traditionally associated with malaria cases in the study area, the township has seen a recent emergence of indigenous malaria cases.

*Implementing partners such as NGO and INGO are working with state VBDC* [vector-borne disease control] *for malaria prevention and control activities*. (Malaria focal person, State Health Department)

*Migrant workers from different parts of Myanmar moved and worked around the goldmines and somewhere here. Subsequently, there were many new worksites and banana plantation sites established which might have resulted in increasing malaria incidence even among children.* (BHS, rural health center)

### IDI theme II: Increasing trend of P. vivax

Myanmar has witnessed a notable shift in the distribution of *Plasmodium* species causing malaria. In Kachin State, *P. falciparum* was historically the dominant species responsible for malaria infections. Recently, however, there has been a steady prevalence of *P. vivax* malaria cases, and at present, nearly all malaria cases in the region are caused by *P. vivax* infections. Township-level healthcare officers noted the increasing trend of *P. vivax* malaria, particularly after 2019.

*In this area*, P. vivax *cases are increasing beginning from 2019 as reported by rural health centers, subcenters, and volunteers. Many patients with* P. vivax *came back from the banana plantation field. Still, we found malaria among local people and children too.* (Malaria focal person, township public health department)

### IDI theme III: Challenges encountered during antimalaria treatment including primaquine

Four subthemes emerged as follows.

#### Insufficient health literacy initiatives

One respondent emphasized the importance of updated treatment guidelines among all health providers in effectively managing malaria infections and ensuring radical cure. Following current guidelines is deemed crucial for the treatment of every malaria patient. Additionally, the respondents highlighted the limitations faced by those who conduct malaria control activities during the COVID-19 pandemic. Onsite activities including state-level coordination meetings with implementation partners, malaria volunteer trainings, refresher courses, and health education sessions were prohibited because of social distancing measures.

*We do not implement regular follow-up schemes for patients with malaria. During COVID-19 pandemic era, we could not organize any malaria health education activity either one by one or in a group because we are all busy with urgent activities like COVID-19 vaccination. Still, we regularly distributed the LLINs [long-lasting insecticide-treated nets] to every malaria-confirmed patient.* (Female BHS, rural health center)

#### Inadequate supply chain management

Most respondents highlighted the need for an effective supply chain for the distribution of malaria commodities, particularly rapid diagnostic kits and antimalarial medicines, in response to the increasing number of malaria cases in recent years. They noted that they could not distinguish relapse cases using the current diagnostic tools.

#### Human resource shortages and constraints

Township-level healthcare officers stated that they usually prescribed complete drugs on a one-time basis without comprehensively communicating the importance of treatment adherence because of time limitations amid workloads. They would usually perform microscopy examinations on hospital-admitted patients and outpatients. Although the township hospital has a malaria microscopy facility, its technician could not cross-check every patient who has undergone a rapid diagnostic test unless a healthcare officer was specially requested. Moreover, because of resource constraints, conducting long-term follow-ups for all malaria cases to identify potential new infections or relapses is not feasible.

*There is no regular follow-up system and detection of relapse in the current malaria surveillance system. Due to political insecurity, supervision of patients can’t be done either.* (Malaria focal person, State Public Health Department)

#### Challenges to patient adherence and compliance

Poor treatment adherence among patients with *P. vivax* malaria was another issue to be prioritized. Typically, healthcare providers would administer the complete drug regimen all at once to every malaria patient. However, they were unsure of the patients’ full treatment compliance, especially for a 14-day PQ course. Some might forget to take the drugs or lose them during the treatment course, especially when their symptoms (e.g., fever) disappear after two to three days of CQ treatment. Other patients had a history of malaria and incomplete treatment two to three months before the current infection.

*We observed that some old patients contracted malaria again after two to three months after the first-time treatment. They have always reported not completing the full drug course in the last treatment. As the area constituted many migrants, a few patients disappeared after the treatment and never reappeared.* (VHV)

### IDI theme IV: Suggestions to improve antimalarial drug adherence among patients with P. vivax

Four subthemes related to the interventions suggested by the participants are as follows.

#### Supervised treatment

Most respondents expressed concern regarding malaria patients’ suboptimal treatment adherence, particularly in cases where long-duration treatment regimens were prescribed. To address this issue, they suggested DOT as a potential solution to improve patient compliance. However, resource constraints such as staffing shortages, budget limitations, and logistical challenges, particularly in rural areas with a high malaria burden, affect the feasibility of intensive DOT implementation by healthcare personnel. Consequently, the respondents proposed a reliable and cost-effective alternative in which DOT is administered by the family members of malaria patients. They emphasized the importance of locally adapting such interventions to individual needs. Furthermore, considering the limited accessibility of mobile phone networks in certain areas, relying solely on follow-up calls for medication reminders was deemed impractical.

*The immediate treatment by a family member may be viable as we applied for organizing mass drug administration activities in the filariasis program*. (Malaria focal person, NMCP)

*We can do some activities such as family member’s DOT and telephone reminder calls or text messages to improve treatment adherence.* (Malaria focal person, township public health department)

#### Shortening of treatment

Although patients with *P. vivax* usually present minor symptoms, the respondents felt that eradicating liver-stage parasites is complicated and requires close supervision. Thus, shortening the current 14-day treatment for *P. vivax* malaria would be a good alternative.

*Shortening the days of primaquine treatment and giving more health education for strengthening the radical cure of* P. vivax *should be implemented. Moreover, volunteer training should be organized to improve their perception of treatment adherence.* (Female, State Health Department, Kachin State)

#### Refresher trainings for volunteers

Because of malaria volunteers’ insufficient provision of detailed information on the importance of drug adherence, their understanding of the potential detrimental consequences of incomplete treatment must be strengthened. Refreshing their knowledge is crucial as it would help them effectively disseminate their acquired knowledge to the wider community and malaria patients.

*Trainings to volunteers should be organized to improve their perception toward treatment adherence*. (Malaria focal person, State Health Department)

#### Behavioral change communication

Village-level healthcare providers concluded that increasing the community’s health awareness of taking a complete treatment course is critical to improving treatment compliance. When prescribing antimalarial medicines, delivering health education using the local language might be effective. The participants proposed an intervention in which health messages are disseminated through sessions organized at religious buildings such as churches or monasteries. They also stated that many villagers preferred receiving health messages through posters and pamphlets including simple facts and fancy pictures or cartoons. If possible, these materials should include contents in the local language.

*We should strengthen the community’s knowledge of malaria treatment so that they would strictly follow our instructions. It would be good if we could conduct health education sessions at churches using pamphlets and posters.* (Male VHV)

### Focus group discussions among community members

Three themes emerged from the FGDs among community members, including malaria-related knowledge, treatment-seeking behavior, and suggestions for further actions within the malaria control program.

### FGD theme I: Malaria-related knowledge

The community members shared their experiences with malaria, with the majority having a history of malaria themselves or in their families within the past two years. They were aware that malaria is caused by mosquito bites and that its cardinal symptom is fever with chill and rigor. However, some respondents chose quick relief by consulting illegal practitioners such as quack doctors. Most community members regularly used bed nets at night except in hot weather and during travel. In recent years, the distributed nets had become large and unusable in the forest. Many people also use the old nets while preserving the newly distributed ones for guests.

*We could not use a net when we traveled as the recently distributed nets were family size which takes too much space. Therefore, we could not use a net if it’s unavailable.* (29-year-old male)

### FGD theme II: Treatment-seeking behavior

The community members discussed the historical and current symptoms of malaria, highlighting a shift from severe to mild. They shared experiences of caring for family members with the disease and noted recurring episodes despite completely taking the drugs prescribed by healthcare professionals. Some participants stopped treatment once their symptoms improved, while others experienced worse symptoms and required hospital referral after oral treatment. One case involved a pregnant woman who had an abortion after taking malaria medication, which led some individuals to become reluctant in taking antimalarial drugs because of concerns about side effects and prolonged usage. Distinguishing malaria fever from other types of fever allowed them to self-diagnose and seek treatment at nearby health centers or from village malaria volunteers.

*We have to take the drugs for many days to overcome malaria infection. However, as I am afraid of drug side effects, most of the time, I took paracetamol to relieve fever. It worked for me well.* (40-year-old male)

### FGD theme III: Suggestions for further actions within the malaria program

Many respondents reported having limited health knowledge, having attained only primary education. Therefore, they preferred to engage in more health education sessions along with the distribution of other malaria-preventive materials such as long-lasting insecticide nets. Several respondents also stated that because malaria control activities primarily relied on healthcare professionals and malaria projects, community members planned to simply follow the instructions accordingly. Overall, they are ambivalent about offering suggestions for the program but remain willing to help healthcare working groups as required.

*I had a chance to attend only grade 5 class, and I do not exactly know malaria-related facts on how to prevent or treat the disease. Therefore, I will be grateful if healthcare providers come to our villages and organize frequent health education sessions*. (46-year-old male)

## Discussion

This study was conducted in a township that is currently a long way from achieving the malaria elimination indicator. The majority of malaria infections were due to *P. vivax.* Relapse and the reactivation of latent hypnozoites have been recognized as the main contributors to the persistence of *P. vivax* transmission [16]. The radical cure of P*. vivax* requires a 14-day PQ treatment. Several interventions have been proposed to improve treatment compliance [17–20]. However, each intervention can vary in effectiveness across areas because of the baseline culture, beliefs, and logistical limitations. Specific interventions should be tailored to increase the chance of successful implementation. This study presented stakeholders’ opinions toward the *P. vivax* malaria burden, challenges and limitations of treatment intervention, and alternative measures to increase adherence to the 14-day PQ treatment.

In this relatively high-malaria-endemic area, the respondents were aware of the disease but still chose to consult illegal medical practitioners such as quack doctors as their first choice because of their perceptions of mild symptoms or familiarity with the disease. This aligns with findings from a study in Lao PDR, where people in highly malarious areas had low perceptions of malaria [21]. In certain areas of Myanmar with no government health centers, quack doctors remain popular and continue to provide healthcare services [22]. Once symptoms such as fever subside, patients with malaria frequently discontinue their prescribed medications. This finding is consistent with a study conducted in Sri Lanka, where a majority of malaria patients were hesitant to continue medication once they felt better [23]. The FGDs revealed that the respondents preferred to receive health education from healthcare providers. In addition, the community promptly participated in health activities led by healthcare providers. Therefore, healthcare providers or malaria volunteers should routinely provide patients infected with *P. vivax* with detailed prescriptions of medicines and health messages regarding the importance of completing their treatment courses.

Ensuring standardized and timely treatment for every confirmed malaria patient is essential for speedy recovery and to prevent complications [24]. However, low literacy surrounding *P. vivax* treatment was the main concern among the healthcare providers participating in this study. Some studies in Myanmar have raised a similar concern about the performance of VHVs [25–27]. Malaria treatment providers, including BHS and VHVs, should be well-prepared to deliver high-quality treatment according to the National Malaria Treatment Guidelines [3]. Therefore, regular refresher sessions on malaria diagnosis and case management, along with field site monitoring visits, are necessary. Adequate stocks of malaria commodities should be maintained through strategic procurement planning.

Research has shown that DOT is an effective intervention to ensure treatment compliance in many diseases including malaria [17]. Considering the relatively high number of reported *P. vivax* cases in the study township, it would be impossible for healthcare providers or VHVs to deliver DOT because of increased workloads and limited human resources. The existing local malaria workforce has highlighted that the DOT strategy is infeasible for malaria treatment unless the incidence of the disease is reduced [28]. A study has shown that sending reminder text messages directly to patients could be an efficient alternative to DOT [29]. However, the limited access to a reliable telecommunication infrastructure, particularly in remote villages with active malaria transmission, poses a challenge for malaria healthcare providers in implementing this approach.

In the study township, the respondents reported an influx of workers migrating to and from worksites including banana plantation fields. Therefore, an alternative measure would involve allocating more malaria volunteers to these worksites to perform follow-up checks with patients who completely take the prescribed drugs. However, its success depends on the number of confirmed patients with malaria and population displacements that result in loss to follow-up [15]. The performance of health volunteers is sometimes associated with the incentive they receive for surviving [3]. An attractive incentive scheme should be based on the magnitude of workloads.

Moreover, many patients with *P. vivax* came from worksites with poor road conditions. Delivering health education to patients and their companions to ensure regular and thorough drug intake has been proven to effectively improve adherence, as demonstrated in the Thailand–Myanmar border area [28]. However, Myanmar consists of numerous ethnic groups and minorities, with Burmese being the major language spoken across the country. Because of language barriers, delivering health messages solely in Burmese may not reach all individuals. As suggested by most respondents, it is important to produce information, education, and communication materials in dual-language formats and involve locals who can communicate health facts in regional dialects during health education sessions. One study recommended using local languages to ensure the effective and efficient dissemination of health-related messages [30].

The current respondents also suggested shortening the course of radical treatment. A multicenter study documented the acceptable efficacy of implementing a seven-day PQ treatment compared with a two-week regimen in reducing *P. vivax* malaria relapse [18]. Similarly, the NMCP may consider piloting this in the study township based on further feasibility assessment of the area and technical support from other organizations such as the World Health Organization.

This study has several strengths and limitations. Because it is the first comprehensive qualitative study that involves malaria stakeholders from all levels of the malaria control program in Myanmar, its findings are valuable and representative. The selected township represents the socio-behavioral conditions of areas where *P. vivax* malaria elimination is challenging. The present findings can be applied to other areas with similar conditions. FGDs were conducted to identify challenges and community needs regarding drug treatment compliance. These results can serve as a reference for new interventions in the National Malaria Strategic Plan. However, this study was conducted in areas where political conflict situations have less impact. The suggested interventions in this study should therefore be adjusted when they are implemented in high-endemic areas.

## Conclusions

Given the emerging trend of *P. vivax* incidence in Myanmar, facilitating patients’ compliance with PQ treatment may facilitate the country’s path toward malaria eradication by 2030. This study provided insights into the challenges to treatment adherence and recommendations for improving it. Interventions include delivering DOT by family members, piloting a shorter PQ regimen, recruiting worksite malaria volunteers in refugee camps or plantation sites, and increasing health education activities specifically to disseminate information on the issue of incomplete or partial treatment among migrants. When possible, health promotion activities must be delivered in local dialects and standardized pamphlets to strengthen the use of long-lasting insecticide-treated nets and drug adherence. Meanwhile, to reduce the malaria caseloads in this township, maintaining a decent surveillance system is essential to promptly detect malaria cases. In addition, each malaria case must receive standard treatment provided by skilled health workers including VHVs. A constant procurement and supply chain system for distributing malaria commodities should also be in place.

## Data Availability

All the data analyzed in this study are already included in the article.

## Abbreviations

BHS: basic health staff
DOT: directly observed treatment
FGD: focus group discussion
G6PD: glucose 6 phosphate dehydrogenase
GMS: Greater Mekong Subregion
IDI: in-depth interview
PQ: primaquine
VBDC: vector-borne disease control
VHV: village health volunteer
